# Age, Sex, and Genetic and Environmental Effects on Unintentional Injuries in Young and Adult Twins

**DOI:** 10.1017/thg.2018.61

**Published:** 2018-11-15

**Authors:** Simo Salminen, Eero Vuoksimaa, Richard J. Rose, Jaakko Kaprio

**Affiliations:** 1 Department of Social Psychology, University of Helsinki, Helsinki, Finland; 2 Institute for Molecular Medicine Finland (FIMM) & Department of Public Health, University of Helsinki, Helsinki, Finland; 3 Department of Psychological and Brain Sciences, Indiana University, Bloomington, IN, USA

**Keywords:** injuries, genetics, environmental effects, twins

## Abstract

The aim of this study was to examine the effects of genetic and environment influences and sex on injury involvement using two sets of Finnish twin data. The younger participants were 955 twins born between 1983 and 1987, aged 20 to 24 years. The older participants were 12,428 twins born between 1930 and 1957, aged 33 to 60 years. Within-twin correlations in monozygotic and dizygotic twins suggested that genetic effects play no role in injury involvement among young twins, but do have some effect at older ages. The results indicated that environmental factors have greater importance in injury involvement than genetic factors in the younger twin data set (FT12), whereas in a middle-aged (33–60 years) twin data set, genetic effects explained about quarter of the variance in injury involvement. Sex was a strong contributing factor, with males being generally more prone to injuries than females.

The first scientific hypothesis explaining accidents was the hypothesis of accident proneness. Greenwood and Woods (1919) proposed that ‘accidents occur to a limited number of individuals who have a special susceptibility to accidents’ (Preface). Accident proneness was presumed to be a relatively stable personality trait. The hypothesis assumed that some people have a dispositional tendency to be involved in accidents (Shaw & Sichel, 1971). Later studies have evaluated this inherent factor from the perspective of personalities of accident-prone people, but results have been inconclusive (Guastello, 1993).

If accident proneness was a dispositional trait, then we would expect to observe a strong genetic influence on injury involvement (Ordonana et al., 2008). Given that behavioral traits generally exhibit moderate to strong familial aggregation, it is of interest to study genetic influences on injury involvement in large family and twin studies. By comparing the similarity of monozygotic (MZ) and dizygotic (DZ) twins, it is possible to assess the relative proportion of genetic and environmental effects on injury involvement. To our knowledge, the effect of heredity on injury involvement among young adults has not been studied.

Previous studies have shown that genetic effects contributed significantly to girls’ unintentional injuries but not to boys’ injuries among Virginia twins (Rowe et al., 2007). However, genetic factors explained only 14% of variance in injury involvement among twins aged 5 years. The present study examined same-sex and opposite-sex twins who were young adults, in addition to reporting previously unpublished results from a survey of middle-aged twins. Together, these provide an estimate of genetic and environmental variance components among adult men and women at different stages of life. In addition to studying the relative importance of genetic and environmental effects, twin samples, including opposite-sex twin pairs, can test whether there are differences between females and males with opposite- and same-sex co-twins. Particularly, investigating differences between females from same-sex and opposite-sex pairs is of interest because earlier literature has indicated that compared to females with a female co-twin, females with a male co-twin may be masculinized in some physiological and/or behavioral traits such as male favoring of mental rotation ability (Vuoksimaa et al., 2010, see Tapp et al., 2011, for a review). The aim of this study was to examine the relative proportion of genetic and environmental effects on injury involvement in two population-based adult (younger and older) twin samples.

## Methods

### FinnTwin12 Data Set

A longitudinal study of five consecutive birth cohorts (1983–1987, FinnTwin 12) of Finnish adolescent twins yielded a database for the investigation of adolescents’ and young adults’ health. The FinnTwin12 study began in September 1994 when the twins were 11-years old (Kaprio et al., 2002). Twins born between 1983 and 1987 and their families (*n* = 5,600) were ascertained from the Finnish Population Register (Kaprio, 2013). For all participants, questionnaire studies were conducted at ages 14, 17, and as young adults (i.e., waves 2, 3, and 4).

From the five consecutive birth cohorts, a subset of 1,035 twin families was formed for the intensive study protocol. This twin-family study investigates mental health and its role in substance use and psychiatric comorbidities, especially in adolescence. All intensive subset twins were interviewed at age 14 (*n* = 1,852) to assess substance use and dependence in adolescence (90% participated in interviews). The fourth wave data collection in 2006–2009 conducted structured psychiatric interviews at an average age of 22 (*SD* 0.8, range 21–24) years for 1,347 twins (73% of target sample). Zygosity was determined using a validated questionnaire method (Sarna et al., 1978), with additional questions for younger twins (Goldsmith, 1991). Zygosity was confirmed from DNA for most of the twins. Of these, 812 were seen in person and the rest were approached by telephone. All subjects participated psychiatric interviews, mailed questionnaires and provided blood or saliva samples (*n* = 1,295) individuals. A total of 955 individuals answered questions on injuries in working life. All participants gave written informed consent before participating in the study. The FinnTwin12 study protocol was approved by the ethical committee of Helsinki and Uusimaa hospital district, Helsinki, Finland and IRB of Indiana University, Bloomington, Indiana. The authors assert that all procedures contributing to this work comply with the ethical standards of the Helsinki Declaration of 1975, as revised in 2008.

Three questions about injuries were presented: (1) How many times have you undergone an examination or received care in an emergency clinic due to injury? The maximum possible value for injuries was ‘99 or more’. (2) How many times have you received medical care due to injuries during the past 12 months, including any you have administered yourself? (3) Where did your last injury happen? (a) at school or when studying; (b) at work; (c) in traffic (including trips to school or work); (d) during sports or physical exercise; (e) at home; (f) during other leisure time; (g) during army duty (compulsory for men, voluntary for women; about 90% of men participated). These questions were included in the interview used at the Finnish Institute of Occupational Health (Kauppinen et al., 2010).

### Older Finnish Twin Cohort

For comparison, we also analyzed twin data from the 1990 questionnaire study carried out as wave 3 of the older Finnish Twin Cohort (Kaprio & Koskenvuo, 2002). The first two questionnaire studies (wave 1 in 1975 and wave 2 in 1981) of the Finnish Twin Cohort were targeted at all available twin pairs consisting of twins born before 1958 and alive in 1974. In 1990, the questionnaire was mailed to twin pairs born 1930–1957, with 12,502 responders (response rate 77%). Of these, 12,428 replied to a single item on injury involvement. It asked whether the respondent had been treated for an injury by a physician or in hospital during the previous 5 years. The four response alternatives were (1) *never*, (2) *once*, (3) *2–4 times*, and (4) *5 or more times*. The responses were analyzed as ordinal measures. Twin zygosity was determined in 1975 or 1981 using a validated questionnaire method (Sarna et al., 1978). Answering and returning the completed questionnaire was considered as consent in the data collections from 1975, 1981, and 1990. The Older Finnish Twin Cohort study was approved by the National Board of Health. During the course of the cohort study, participants have been repeatedly informed about the study and that they may withdraw from it whenever they wish.

### Statistical Analyses

We used chi-square tests to compare the prevalence of injury between men and women and between twins from same-sex and opposite-sex pairs. Family structure (twins within families) was taken into account in these analyses. We calculated tetrachoric (dichotomic variable in FinnTwin 12) and polychoric (ordinal variable in the Older Finnish Twin Cohort) correlations in twin pairs to compare the similarity of MZ and DZ pairs in injury involvement.

In the twin design, it is possible to decompose the phenotypic variance into additive genetic (A), common environmental (C), and unique environmental (E) components. The model consisting of these components is called the ACE model. The estimation of genetic and environmental effects is based on the assumption that A has a correlation of 1.0 in MZ twins (who are generally identical), whereas the correlation is 0.5 in DZ twins (who share on average 50% of their segregating genes and have a genetic resemblance similar to non-twin siblings). C effects denote all environmental effects that make twins within a pair similar to each other and therefore these effects correlate 1.0 both in MZ and DZ twins. E effects are uncorrelated and denote all environmental effects that make twins within a pair different from each other. In addition, measurement error is included in the E effects. Analyses were performed using the maximum-likelihood-based, structural equation modeling software Mx. In the univariate model, we first estimated A, C, and E effects. In subsequent models, we constrained A and C effects to zero and tested whether these more parsimonious models fit the data or if fixing this parameter provides a deterioration of the model compared to the full ACE model. Model comparisons were based on the likelihood-ratio chi-square test, which is calculated as the change in -2 log likelihood (-2LL) from the ACE model. In these models, a *p* value <.05 indicates significant deterioration of the AE or CE models against the full ACE model.

## Results

### FinnTwin12 Data

One-fourth (24%) of the respondents who answered the injury questions had been involved in an injury requiring medical care during the past 12 months. Most of the injured had suffered only one injury (16% of the respondents), 4.5% were involved in two injuries, and 3.1% in three or more injuries.

Men reported injuries more often than women. Among twins from same-sex pairs, males were significantly more often involved in injuries (32.7%) then females (16.1%), *F*(1, 506) = 33.15, *p* < .001). If twins were from opposite-sex pairs, males reported more injuries (27.1%) than females (22.5%), but this difference was not statistically significant, *F*(1, 219) = 1.35, *p* = .25. The injury rate was significantly higher (22.5%) in females with a male co-twin compared to females with a female co-twin (16.1%), *F*(1, 462) = 3.88, *p* < .05). In males, there was no statistically significant difference between males with a male co-twin (32.7%) and males with a female co-twin (27.1%), *F*(1, 435) = 1.94, *p* = .16.

One-third of injuries occurred during sports and physical exercise. One out of four injuries was an occupational injury and one-fifth a leisure-time injury. Men reported more often injuries occurring during sports and physical exercise, whereas women had home injuries more often than men (Fisher exact test = 0.0015) (Table 1).

**TABLE 1:** Place of Last Injury by Gender

		Gender,	
	Male	% Female	
Place of injury	(*n* = 139)	(*n* = 77)	Total
At school	1.4	3.9	2.3
At work	23.1	23.4	23.2
In traffic (to school or work)	5.0	10.4	6.9
During sports and exercise	41.1	24.6	35.3
At home	4.3	15.6	8.3
During leisure time	20.1	22.1	20.8
During army duty	5.0	0.0	3.2

When we analyzed pairwise similarities for the injury variable (for all injuries: 0 = no injury, 1 = injury), tetrachoric correlations in MZ (0.13, *ns*) and DZ twins (0.34, *p* < .05) indicated that genetic factors had no role in injury involvement. However, the standard errors of both twin groups were rather large (0.12 in MZ and 0.12 in DZ twins, respectively). Both twins were injured in 8.6% of pairs, only one twin in 31.0% of pairs, and neither twin in 60.4% of pairs (chi-square = 13.36, *df* = 1, *p* < .001, when testing injury involvement in pairs). Because the MZ within-pair correlations were non-significant, we did not perform structural equation modeling in this sample (i.e., a non-significant MZ within-pair correlation indicates that all the variance is explained by E effects; i.e., not shared by twins). Tetrachoric correlations in MZ and DZ twins by sex were MZ males = 0.01, standard error (*SE*) = 0.16; MZ females = 0.13, *SE* = 0.19; DZ males = 0.40, *SE* = 0.16; DZ females = 0.23, *SE* = 0.20.

In the next stage, we limited the analysis to those twins who currently lived apart (*n* = 541). The DZ twin correlation (0.28) remained higher than that of MZ twins (0.04), indicating negligible genetic effects on injury involvement. Standard errors were the same (0.13) for both groups. The number of twins living together (*n* = 414) was too low for the analysis (no full DZ male pairs involved in injuries, and only one full pair in MZ and DZ women involved once in an injury).

### Older Finnish Twin Cohort

In the older twin data, men (*n* = 5,664) also reported more injuries than women (*n* = 6,764): 56% of men and 75% of women reported no injuries treated by a physician or in a hospital in the prior five years. Correspondingly, 25% of men and 18% of women reported one treatment episode, 17% of men and 5% of women reported 2–4 injuries that required treatment, and 2.6% of men and 1.4% of women reported five or more such injuries. The gender difference is highly significant (*p* < .001).

There were a total of 1,531 MZ and 2,945 DZ pairs with injury data. The polychoric correlation for liability to injury was slightly higher in MZ pairs (females: *r* = 0.24, *SE* = 0.055; males: *r* = 0.196, *SE* = 0.054) than in DZ pairs (females: *r* = 0.134, *SE* = 0.042; males: *r* = 0.142, *SE* = 0.038). The best-fitting model for females was an AE model, with a heritability estimate of 25% (Table 2). For men, both AE and CE models fit well, with heritability and shared environmental estimates of 22% and 16%, respectively, with the remainder attributed to non-shared environmental effects (Table 2).

**TABLE 2:** Model-Fitting Statistics

Model	Δ-2ll	Δ*df*	*p*
Women			
ACE	–	–	–
AE	0.246	1	.620
CE	7.342	1	.007
E	84.436	2	<.001
Men			
ACE	–	–	–
AE	1.754	1	.185
CE	1.319	1	.251
E	50.139	2	<.001

Δ-2ll = change in minus two log-likelihood, Δ*df* = change in degrees of freedom. The fit of the AE, CE, and E models are tested against full ACE model. A *p* value less than .05 indicates a significantly poorer model fit.

## Discussion

Utilizing two population-based samples of Finnish twins at different ages, we aimed to examine the magnitude of genetic effects in injury involvement in young adulthood and in a sample of older twins. The results indicated that genetic factors played at most a minor role in injury involvement. The analysis of twins living apart in the younger data set further supported this conclusion. This result is consistent with the study of unintentional injuries of British children (Ordonana et al., 2008).

Men were involved in injuries more often than women. This is true for all types of injuries except home injuries, in which women were involved more often. These results are in line with earlier studies (e.g., Salminen, 2004). Men were involved in military service, which is voluntary for women. Most of the injuries in the Finnish army were of the same kind as sports injuries; for example, a sprained ankle.

A novel aspect of the present study was the possibility of evaluating ‘the masculinization effect’, whereby the females from opposite-sex twin pairs had had a higher prevalence of injury (23%) during the last year than females from same-sex pairs (16%). Earlier, a replicated masculinization effect was observed in, for example, male-favoring mental rotation ability (Vuoksimaa et al., 2010). Possible masculinization of females with a male co-twin may result from either prenatal exposure to testosterone or from postnatal environmental effects (e.g., exposure to typical male play and toys) (Tapp et al., 2011). Male twins were more often involved in injuries than female twins. This fact also dominates the observation that boys with a female co-twin have similar injury rate to boys with male co-twin. However, if a female twin had a male co-twin, this appeared to increase her injury involvement. Although our results indicated that a male twin may influence the behavior of his female co-twin, we cannot conclude whether such masculinization effects are prenatal or postnatal in origin. The finding may also be due to chance and needs to be replicated in a larger study.

### Strengths and Limitations

As far as we know, this is the first study to investigate the genetic and environmental influences on young adults’ injuries. Participants of the FinnTwin12 study were in the age group (20–24 years of age) that is most often involved in injuries (Salminen, 2004). The strength of this study was the determination of twin zygosity, which is based on multiple, polymorphic genetic markers; in the older twins, zygosity was determined using a questionnaire approach that has been validated using genetic markers (Sarna et al., 1978). Another strength was the population-based samples with the high participation rates.

The main limitation of this study is that the collected injury information comes from the participants retrospectively. In FinnTwin12, participants were asked to report their injuries during the last 12 months, which is the usual follow-up time in injury research. This reference time is too long, as people forget about 30% of their injuries within 1 year (Elander et al., 1993). However, too short a reference time may mean too small a number of injuries in the data. People at this age do different things: some are full-time students, others full-time workers, many are both working and studying, and some are in military service. This might make it more difficult to detect genetic effects among young adults.

When we asked about injuries during the past 5 years in the older cohort, we had only one measure of injury involvement, namely having been treated by a physician or in hospital. In the analysis, we have some evidence for a genetic component with a much larger sample size. Nonetheless, in men, we could not distinguish between genetic and shared environmental effects. We also note that the proportion of twin pairs concordant for injury involvement was low. Very large samples are needed to detect genetic effects of a small magnitude in case of dichotomy or ordinal variables (see, e.g., Medland et al., 2006). Future studies should use a larger sample size when studying the genetic and environmental effects on injury involvement. These can be obtained primarily through the use of register information or by pooling several data sets from many twin cohorts.

Our injury data was based on self-reporting, which is the most often-used way to measure injuries. The subjects may remember their injuries in different ways; however, comparisons have usually shown a rather good correspondence between self-reported injuries and company records (Salminen et al., 2009). When examining different kinds of injuries, self-reporting is the best way to collect data regarding all injuries; this also includes minor injuries.

## Conclusions

Our results suggested that genetic effects play at most a very modest role in injury involvement in adults. Moreover, in young adulthood, genetic factors appear to have no role in injury involvement. In a much larger sample of middle-aged adults, we observed some evidence for a heritability of injury involvement (additive effects 22%). Nonetheless, considering the familial effects in men, we could not distinguish between genetic and shared environmental effects.

Males were injured more often than females in all types of injuries except home injuries. Moreover, females with a male co-twin were more often involved in injuries than females with a female co-twin.

The results indicated that environmental factors have greater importance in injury involvement than genetic factors. Injury prevention should be focused on making the environment safe for all.

## Contributorship

Kaprio, Rose, and Vuoksimaa designed the study and organized the gathering of data. Salminen created the questions related to the injuries in the FinnTwin12 study and written a first version of manuscript. All authors have commented critically on the manuscript and approved the final version.
